# The role of dysregulated ghrelin/LEAP-2 balance in anorexia nervosa

**DOI:** 10.1016/j.isci.2023.107996

**Published:** 2023-09-22

**Authors:** Chloé Tezenas du Montcel, Philibert Duriez, Jingxian Cao, Nicolas Lebrun, Nicolas Ramoz, Odile Viltart, Philip Gorwood, Virginie Tolle

**Affiliations:** 1 Université Paris Cité, UMR-S 1266 INSERM, Institut de Psychiatrie et Neuroscience de Paris (IPNP), 75014 Paris, France; 2 Clinique des Maladies Mentales et de l’Encéphale, GHU Paris Psychiatrie et Neurosciences, Hôpital Sainte-Anne, 75014 Paris, France; 3 Université de Lille, SCALab - Sciences Cognitives et Sciences Affectives, UMR CNRS 9193, PsySEF département, 59653 Lille, France

**Keywords:** Health sciences, Medicine, Psychiatry, Endocrinology, Natural sciences, Biological sciences, Nutrition

## Abstract

LEAP-2 is a ghrelin antagonist with an anorexigenic drive. This study investigates the evolution of plasma ghrelin and LEAP-2 concentrations in 29 patients with anorexia nervosa (AN) before and after refeeding and compares it to physiological adaptations during fasting in healthy controls or to mouse model of chronic food restriction and refeeding. Acute and chronic food restriction decrease LEAP-2 and increase ghrelin concentrations in both humans and mice, while patients with AN displayed higher ghrelin and LEAP-2 concentrations before than after refeeding (p = 0.043). After 6 months follow-up, patients with unstable weight gain (n = 17) had significantly decreased LEAP-2 concentrations after refeeding (p = 0.044), in contrast to patients with stable weight gain (n = 12). We provide evidence that the ghrelin/LEAP-2 system is not regulated according to the nutritional status in AN, in contrast to what is physiologically expected when coping with food restriction.

## Highlights

• Low LEAP-2 is a biomarker of undernutrition in physiological conditions
• Patients with AN display higher LEAP-2 levels in undernourished compared to refed state
• Abnormal LEAP-2 regulation during refeeding is associated with unstable remission

## Graphical abstract

**Figure:**
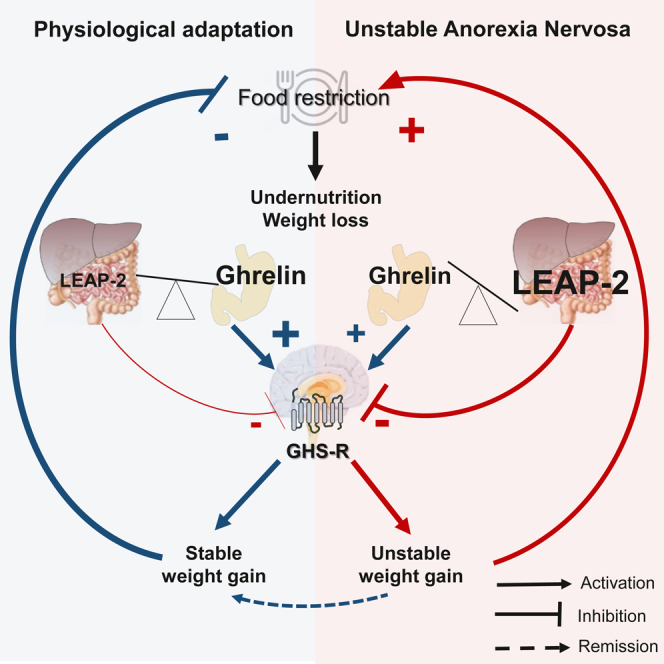

## Teaser

Health sciences; Medicine; Psychiatry; Endocrinology; Natural sciences; Biological sciences; Endocrinology; Nutrition

## Introduction

Eating is a complex behavior implicating metabolic sensors of energy balance, sensory inputs, environmental factors such as food availability, emotional and reward-associated processes.^1^ Eating regulation relies on crucial neuroendocrine feedback loops between the hypothalamus, brain stem and peripheral organs such as the pancreas, adipose tissue and gastrointestinal tract that permit the adaptation of food intake to energy needs. Maladaptive eating, a core feature of eating disorders, can be described as non-homeostatic food intake that wouldn’t be correlated with energy needs. Anorexia Nervosa (AN) is a complex eating disorder characterized by compulsive self-restriction of food intake leading to drastic weight loss affecting mainly young women with a mean sex ratio of 9 females for 1 male.^2^ Recent genome wide association studies demonstrated genetic associations of both metabolic and psychiatric traits with AN, explaining that AN is now considered as a “metabo-psychiatric disorder.”^3^ Indeed, multiple evidence suggest an uncovered role of metabolic sensors, such as regulators of appetite, in the pathophysiology of AN.^4^^,^^5^^,^^6^^,^^7^ Among these sensors, acyl-ghrelin (AG) is an active form of ghrelin, a pleiotropic hormone secreted by gastric cells, and an endogenous agonist of the Growth Hormone Secretagogue Receptor (GHSR).^8^ AG is an orexigenic peptide with an ultradian pattern of secretion involved in meal anticipation and initiation in healthy humans and rodents.^9^^,^^10^^,^^11^^,^^12^ One of the main brain targets where AG acts to induce food intake is the hypothalamic arcuate nucleus, where it activates GHSR-expressing NPY/AgRP neurons.^13^ Through its Growth Hormone (GH) secretagogue actions, AG also has a major role in survival during undernutrition in rodents to stabilize blood glucose and body temperature.^14^^,^^15^ Previous studies showed elevated AG levels in undernourished patients suffering from AN and in mouse models of chronic undernutrition, defining ghrelin as a metabolic sensor of chronic undernutrition.^11^^,^^16^^,^^17^^,^^18^ However, despite high levels of this orexigenic hormone, patients suffering from AN do not eat sufficiently to reduce undernutrition suggesting a possible ghrelin-resistance mechanism.

LEAP-2 (Liver Expressed Antimicrobial Peptide 2) is a recently discovered GHSR antagonist and inverse agonist produced in the liver and gastrointestinal tract that opposes ghrelin actions on GH secretion, blood glucose and food intake.^19^^,^^20^^,^^21^^,^^22^^,^^23^ A clinical study by Mani et al. showed elevated LEAP-2 concentrations in patients with increased body mass index (BMI), specifically when the BMI reached over 40 kg/m^2^, and LEAP-2 levels decreasing with weight loss.^24^ Similarly, preclinical studies showed a modulation of LEAP-2 levels with nutritional status being increased in a model of diet-induced obesity but decreased after a 24h-fast.^21^^,^^24^ However, no previous study explored the role of the ghrelin/LEAP-2 balance as a marker of appetite regulation and metabolic adaptions in response to chronic undernutrition. Indeed, increased levels of LEAP-2 may modify motivation to eat in patients suffering from AN and participate in maintenance factors of the disorder.

We here tested if female patients suffering from AN exhibit abnormal LEAP-2 regulation in a study that explores for the first time how LEAP-2 is regulated during chronic food restriction and after refeeding in female mice and in female patients suffering from AN.

## Results

### AG/LEAP-2 ratio is a sensor of chronic food restriction and refeeding in female mice

**Figure 1:**
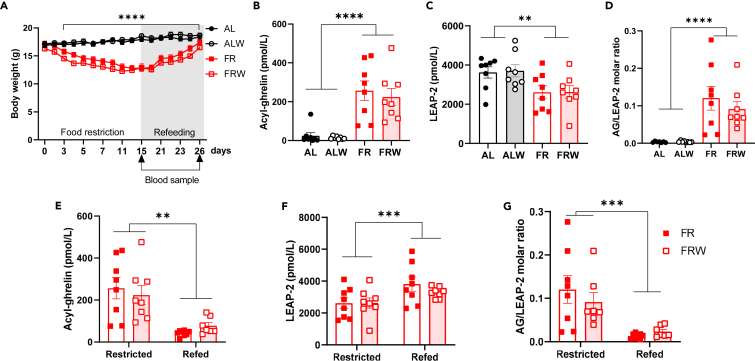
AG/LEAP-2 ratio is a sensor of chronic food restriction and refeeding in female mice Food-restricted mice of both FR and FRW group exhibited significant weight loss compared to *ad libitum* fed controls (A) but progressive refeeding over 10 days restored their body weight. Chronic food restriction was associated with increased plasma levels of acyl-ghrelin (B) and decreased levels of LEAP-2 (C) independently of running wheel in home-cage. AG/LEAP-2 ratio was higher in FR and FRW mice compared to *ad libitum* fed controls with no effect of the running wheel. Progressive refeeding lead to a significant decrease in AG plasma levels (F) and increase in LEAP-2 levels (G) leading to decreased AG/LEAP-2 molar ratio in refed FR and FRW mice. n = 8 animals per group. After refeeding, blood samples of the FRW group were assayed for AG in 7 mice only because of insufficient blood volume extracted at sacrifice for one animal. Data are presented as mean ± SEM plus individual values and were analyzed using three-way ANOVA (A), two-way ANOVA (B–D) or RM ANOVA (E–G) as described in Table S1. ∗∗p < 0.01, ∗∗∗p < 0.001, ∗∗∗∗p < 0,0001. AG: Acyl-ghrelin. LEAP-2: Liver-expressing Antimicrobial Peptide.

To assess the impact of long-term undernutrition combined with physical activity on AG/LEAP-2 ratio, we measured AG and LEAP-2 plasma concentrations after 14-day of food restriction (FR) followed by 10-day of progressive refeeding in female mice exposed to FR (n = 8) or to FR associated with running wheel activity (FRW, n = 8) (Figure 1). We designed this progressive refeeding to mimic specialized care that often offer progressive adapted food rehabilitation programs for patients. All details concerning ANOVA data are provided in Table S1. Body weight rapidly decreased in food-restricted mice and stabilized around 75% of *ad libitum* body weight around day 13 (Time p < 0.001, Food Restriction p < 0.001, Running ns, Time x Food Restriction p < 0.001, Time x Running p < 0.001, Food Restriction x Running ns, Time x Running x Food Restriction ns, Figure 1A). Both FR and FRW mice exhibited increased AG concentrations (Food restriction p < 0.001, Running ns, Food Restriction x Running ns, Figure 1B), decreased LEAP-2 concentrations (Food Restriction p = 0.002, Running ns, Food Restriction x Running ns, Figure 1C) leading to a 10-fold higher AG/LEAP-2 ratio (Food restriction p < 0.001, Running ns Food Restriction x Running ns, Figure 1D) in restricted mice compared to *ad libitum* fed controls. AG/LEAP-2 molar ratio was negatively correlated with the percentage of *ad libitum* body weight calculated in FR and FRW mice on day 14 (r^2^ = 0.32, p = 0.021, data not shown). After 14 days of FR, we started a progressive quantitative increase of food over 10 days and mice reached their baseline body weight on Day 25. Food-restricted animals housed with a running wheel displayed anticipatory wheel running activity as previously described^25^ and progressive refeeding lead to a progressive quantitative decrease in food anticipatory activity (data not shown). After 10 days of refeeding, mice exhibited decreased concentrations of AG (Refeeding p < 0.001, Running ns, Refeeding x Running ns, Figure 1E) but increased concentrations of LEAP-2 (Refeeding p < 0.001, Running ns, Refeeding x Running ns, Figure 1F) compared to their food-restricted state on Day 15. Therefore, refeeding was associated with a significant decrease of AG/LEAP-2 ratio (Refeeding p < 0.001, Running ns, Refeeding x Running ns, Figure 1G). We found no statistically significant effect of running wheel activity on AG or LEAP-2 levels nor interaction with nutritional status in *ad libitum*, food-restricted or refed animals.

Previous findings described a fasting-induced decrease of LEAP2 and reciprocal increase of AG, both of which were also reversible upon refeeding.^21^^,^^24^ We measured the changes in AG and LEAP-2 concentrations through a 48h-fasting paradigm followed by 48 h *ad libitum* refeeding in seven weeks old female mice. Acute fasting led to a significant decrease in body weight and mice entirely recovered after 48h of *ad libitum* access to food (p < 0.001, Figure S1A). During fasting, mice exhibited significantly increased levels of AG (p = 0.022, Figure S1B) and decreased levels of LEAP-2 (p = 0.003, Figure S1C) compared to refed state. AG/LEAP-2 molar ratio was 4 times higher in fasted mice (Figure S1D) and was highly correlated with weight loss in fasted mice (r^2^ = 0.805, p = 0.006, data not shown). Similar data were obtained in a similar protocol using male mice (data not shown). Therefore, both fasting and long-term food restriction led to reduced LEAP-2 levels that normalized upon refeeding.

### AG/LEAP-2 balance is not correlated with nutritional status in patients with anorexia nervosa

We compared preclinical data in female rodents to clinical data from a fasting and refeeding protocol in five healthy female volunteers to confirm that AG and LEAP-2 regulations were similar in mice and humans. Participants were five healthy females aged between 20 and 29 years old, all with normal BMI between 18.8 and 22.8 kg/m^2^ (mean ± SEM = 20.40 ± 0.73 kg/m^2^). AG and LEAP-2 levels were measured following a 15-h fast, and after participants were refed. Refeeding lead to a significant increase in blood glucose levels (p = 0.044, Figure S1E), a decrease in plasma levels of AG (p = 0.019, Figure S1F). LEAP-2 levels increased in four participants, while one exhibited an opposite pattern of regulation (ns, Figure S1G). Finally, AG/LEAP-2 molar ratio significantly decreased with refeeding in healthy female volunteers (p = 0.034, Figure S1H). Taken together, our data suggest that AG and LEAP-2 levels undergo similar regulation patterns in female mice and women after acute fasting and refeeding.

**Figure 2:**
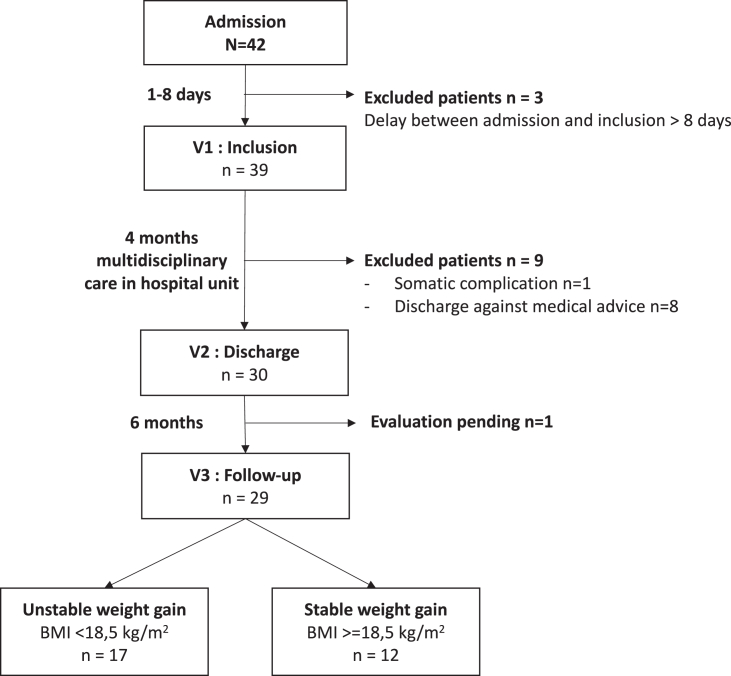
Flow chart of the longitudinal study in patients suffering from AN 42 patients following the inclusion criteria were admitted in the inpatient program so far. Among them, 39 patients were initially included in the study and 3 patients had to be excluded because the delay between admission and inclusion exceeded 8 days. During hospitalization, 9 patients were excluded because they dropped out of the inpatient program, and one was excluded because of somatic complications. On the remaining patients, 29 out of 30 went through the follow-up visit 6 months after discharge.

**Figure 3:**
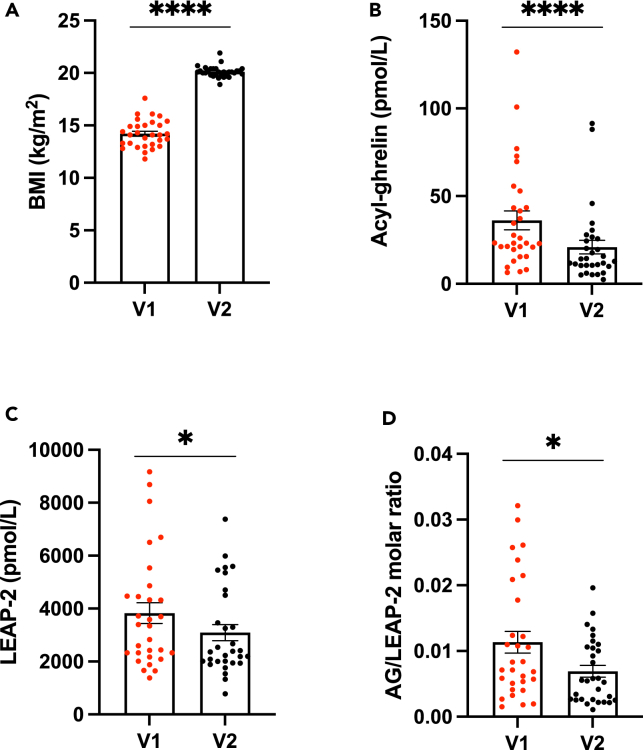
Dysregulated AG/LEAP-2 balance in patients with AN BMI (A) increased with refeeding during hospitalization in 30 patients with AN. AG levels (expressed in pmol/L, B) were significantly decreased with refeeding. LEAP-2 levels were also significantly decreased with refeeding (C). AG/LEAP-2 molar ratio (D) decreased after refeeding compared to undernourished state. Data are expressed as mean ± SEM plus individual values and were analyzed using paired Student’s *t* test: ∗p < 0.05, ∗∗∗∗p < 0.0001. BMI: Body mass Index (kg/m^2^), AG: Acyl-ghrelin. LEAP-2: Liver-expressing Antimicrobial Peptide-2. V1: undernourished state at admission; V2: refed state at discharge.

We then explored changes in AG and LEAP-2 plasma levels in an ongoing longitudinal study that will include 125 female patients suffering from AN with repeated evaluations at various nutritional status. 42 patients were already admitted for an inpatient program offering multidisciplinary care combining nutritional support, individual and group psychotherapy. Patients were included between 1 and 8 days after their admission at hospital in a multidisciplinary intensive food rehabilitation program (V1) and after four months of hospitalization (V2), 3 patients were excluded because of late inclusion. After inclusion, 9 patients were excluded from the preliminary analysis because they did not go through the full hospitalization program, mainly declaring discharge against medical advice (8 patients), while 1 patient was excluded for somatic complications as presented in the flow diagram (Figure 2). These patients were excluded from subsequent analyses. Demographic and clinical data are summarized in Table S2. All patients attained a normal body mass index (BMI) at discharge (V2) and their BMI had significantly increased with refeeding (p < 0.001, Figure 3A). At discharge, patients had lower symptom severity on the EDI-2 scale (p < 0.001) and a lower drive for physical activity on the EAI scale (p = 0.015). Plasma concentrations of AG were elevated at V1 but significantly decreased with refeeding, consistent with previous findings (p < 0.001, Figure 3B). Unexpectedly, plasma LEAP-2 concentrations were significantly higher at V1 and decreased following refeeding (p = 0.048 Figure 3C). The AG/LEAP-2 molar ratio was lower in patients after refeeding compared to the undernourished state (p = 0.005, Figure 3D) but it was not correlated with the patients’ BMI (r^2^ < 0.001, p = 0.897, data not shown). We found a positive correlation between LEAP-2 plasma concentrations at inclusion and LEAP-2 concentrations at discharge (r^2^ = 0.22 p = 0.003, data not shown). However, there was no correlation between LEAP-2 levels at inclusion and other clinical evaluations (age, BMI, duration of illness, EDI-2 and EAI).

### Higher LEAP-2 levels at admission than after discharge is associated with unstable remission

**Figure 4:**
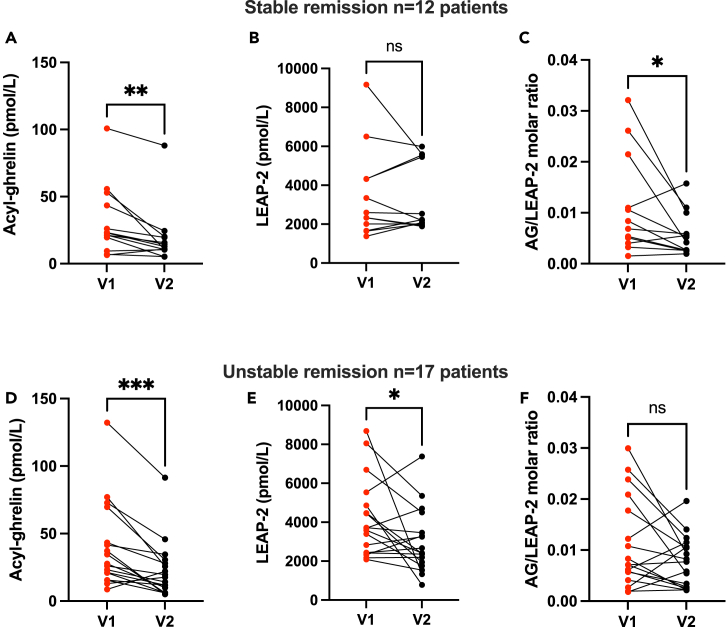
Higher LEAP-2 levels at admission than after discharge is associated with unstable remission AG/LEAP-2 evolution with refeeding in patients with stable (n = 12, BMI>18.5 kg/m^2^, A–C) and unstable remission (n = 17, BMI<18.5 kg/m^2^, D–F) 6 months after discharge. AG levels decreased after refeeding in stable (A) and unstable (D) patients. LEAP-2 levels remained stable during refeeding in patients with stable remission at 6 months (B) but significantly decreased in patients with unstable remission (E). The AG/LEAP-2 molar ratio decreased with refeeding in patients with stable remission (C) but did not significantly differ between before and after refeeding in patients with unstable remission (F). Data are expressed as individual values and were analyzed using paired Student’s *t* test or Mann-Whitney: ∗p < 0.05, ∗∗p < 0.01, ∗∗∗p < 0.001. AG: Acyl-ghrelin. LEAP-2: Liver-Expressing Antimicrobial Peptide-2.

To explore factors associated with remission in AN, we performed an analysis on patients that have already been evaluated 6 months after discharge. As the study is still ongoing, 29 patients out of 30 went through the follow-up visit so far, with, up to now, no lost to follow-up. Among them, 12 were diagnosed with stable remission (i.e., BMI≥ 18.5 kg/m^2^) and 17 with unstable remission (i.e., BMI<18.5 kg/m^2^). Patients of both groups had similar age, duration of the disorder and EDI-2 score at inclusion and at discharge (Table S3). BMI were similar between groups at inclusion but, while weight gain during the program was similar between groups, patients with stable remission surprisingly exhibited a higher BMI at discharge compared to patients with unstable remission (p = 0.042). AG levels decreased during refeeding in both subgroups (AG levels V1 versus V2 in “Stable” p < 0.01, in “Unstable” p < 0.001, Figures 4A and 4D). Interestingly, we found that patients with unstable remission displayed higher LEAP-2 levels at inclusion compared to patients with stable remission although the difference was unsignificant (p = 0.131). Moreover, LEAP-2 change with refeeding was different between subgroups. LEAP-2 plasma levels remained stable in patients with stable remission but decreased in patients with unstable remission (LEAP-2 levels V1 versus V2 in “Stable” p = 0.640, in “Unstable” p = 0.044 Figures 4B and 4E). Finally, we found no significant difference in AG/LEAP-2 ratio between subgroups at both time-points. However, the AG/LEAP-2 ratio was significantly decreased with refeeding in the stable remission group sample of patients (AG/LEAP-2 molar ratio V1 versus V2 in “Stable” p = 0.043, Figure 4C) whereas no significant changes in AG/LEAP-2 ratio with refeeding in the unstable remission group (p = 0.078, Figure 4F) was observed. Indeed, we found very inconsistent patterns of evolution of AG/LEAP-2 ratio in the “Unstable” subgroup with some patients exhibiting decreasing AG/LEAP-2 molar ratio during refeeding and other having increasing ratio.

## Discussion

Taken together, these data suggest that patients with AN exhibit greater plasma concentration of LEAP-2 before than after weight recovery, inconsistently with preclinical data in which food restriction was associated with low LEAP-2 levels. Moreover, this decrease was greater in a subgroup of patients who exhibited unstable remission 6 months after discharge. We could hypothesize that patients with AN exhibit a maladaptive metabolic response of the ghrelin/LEAP-2 system to food restriction and refeeding that could contribute to unstable remission.

Various animal models have been designed to explore maladaptive eating in the field of AN, the most used being the Activity-Based Anorexia (ABA) in which rodents are exposed to time-restricted access to food and access to a running wheel in their home-cage.^26^ Only acute fasting was previously used to explore LEAP-2 variations with undernutrition showing low LEAP-2 levels when animals are fasted compared to when they are sated.^21^^,^^24^ Here, we used a progressive food restriction paradigm that mimics physiological adaptations to chronic energy deprivation observed in patients suffering from AN with reduced fat oxidation, low leptin levels, high plasma ghrelin levels and disrupted estrous cycle.^25^ In our study, we found similar adaptations to fasting or long-term food restriction in mice that decreased LEAP-2 levels that then increased with refeeding. Our data from a small sample of healthy women show that a similar regulation of LEAP-2 with refeeding following an acute fast occurs in humans and in mice. This regulation is consistent with the need for an orexigenic drive by ghrelin in situations of food deprivation to enhance food intake.

Our original intention was to evaluate physiological regulations of those metabolic sensors of appetite and food intake in patients with AN who display decreased spontaneous food intake in situations of chronic energy deprivation. The first interesting finding is that LEAP-2 levels are 10 times greater than AG in food-restricted mice leading to low AG/LEAP-2 ratio although these animals do not display inappropriate feeding behavior when food is delivered. Therefore, the AG/LEAP-2 ratio remains a partial indicator of feeding regulation. Secondly, LEAP-2 levels are increased during the undernutrition state in patients with AN and decreased with weight gain, an evolution that is not reciprocal to ghrelin levels as it is in preclinical models or in human models of acute fasting. No other study explored the LEAP-2 regulation in AN but Mani et al. measured changes in LEAP-2 levels in obese patients before and after weight loss from bariatric surgery.^24^ Interestingly, they showed similar results in patients suffering from obesity and in mice fed with high-fat diet (i.e., increased plasma levels of LEAP-2). Bariatric surgery lead to significant weight loss and significant decrease in LEAP-2 levels. In AN, we show that the changes of LEAP-2 levels are different from physiological regulation. The physiological regulation of the ghrelin system should tend toward lower LEAP-2 level but we show higher LEAP-2 levels in undernourished state that could indicate an abnormal adaptation in these patients.^27^ This decrease during refeeding appears to be greater in patients with unstable remission after discharge although these patients exhibited no other clinical specificities compared to stable patients. Therefore, LEAP-2 levels at admission and its evolution during refeeding may be a new indicator of appetite regulation in eating disorder that is not identified by currently used clinical or biological evaluations. Indeed, patients with unstable remission have no negative predictive markers such as long duration of illness, low BMI at discharge, severity of eating disorder symptomatology, nor exercise addiction.^28^ Changes in LEAP-2 levels could give additional information in order to predict unstable remission after discharge. In a recent study led on healthy men, Hagemann et al. showed that LEAP-2 infusion decreased spontaneous food intake during an *ad libitum* meal.^29^ In this study, authors show that LEAP-2 levels remain stable before, during and after a meal although AG levels decrease after refeeding. These results can be compared with our data in which LEAP-2 levels are not significantly modified before or after a meal in healthy women. However, our data show inter-individual variations and most participants displayed increased LEAP-2 levels in the postprandial compared to the preprandial state, which seems consistent with preclinical studies on the anorexigenic potential of LEAP-2. A recent review of Lu et al. described how LEAP-2’s inhibition of ghrelin’s activity could be a potential target to limit food intake in obesity.^30^ In AN, it can be hypothesized that high LEAP-2 levels could inhibit the orexigenic drive of ghrelin and lead to decreased appetite and motivation for food. Conversely, Ragland et al. showed that a two-weeks low-calorie diet decreased fasting LEAP-2 levels in female patients suffering from obesity.^31^ Interestingly, they explored the role of physical activity associated with diet and showed that while patients exposed to low-calorie diet only exhibited a glucose-induced LEAP-2 rise after a meal, patients exposed to diet and physical exercise had a blunted LEAP-2 rise. Our rodent data seem to suggest similar result with a slightly higher decrease in AG and LEAP-2 rise after refeeding in FR compared to FRW mice although the difference didn’t reach statistical significance. Physical activity could modify LEAP-2 changes with nutritional status, but further study should address to this matter specifically.

Motivational drive is crucial in physiological eating and reward-oriented behavior have been extensively explored in AN. Reward alterations are considered as a core feature of the disorder among which a positive value associated with thinness and an increased capacity to delay reward.^32^^,^^33^ A recent publication by Bernadoni et al. showed a correlation between plasma levels of ghrelin and sensitivity for delayed gratification in patients suffering from AN, revealing an association between metabolic sensing and reward-oriented behavior.^34^ Multiple evidence show the role of the ghrelin system in motivational and reinforcing aspects of food intake through an activation of mesocortico-striatal circuitry mediating the rewarding value of hedonic food.^5^^,^^18^^,^^35^^,^^36^^,^^37^ Further studies should explore the role of LEAP-2 on motivation and reward-oriented behavior during long-term food restriction and refeeding. This finding emerged from a pilot analysis and should only be considered as such but still gives encouraging evidence that LEAP-2 change could be considered as a potential biomarker of remission. Our results constitute a first glance to an ongoing work in which 125 patients suffering from AN will be included that will allow us to further explore the dynamical changes and metabolic adaptation to refeeding throughout and after hospitalization. Identifying biomarkers of stable weight gain remain a crucial challenge in the field of AN to offer individualized targeted interventions for patients with higher risk of relapse after hospital discharge.

### Limitations of the study

The main limitation of this study is the absence of the control group in the longitudinal follow-up study focusing on long-term food restriction. No healthy controls could be exposed to long-term fasting for obvious ethical considerations therefore we used a preclinical model to elucidate the physiological regulations of AG and LEAP-2 to long-term quantitative food restriction. Data from the acute fasting/refeeding protocol in healthy volunteers are consistent with the existing literature and plead in favor of an absence of interspecies differences in the regulation of AG and LEAP-2 plasma concentrations in response to refeeding.^24^ We provide a longitudinal observational study and results suggest an abnormal ghrelin/LEAP-2 regulation in undernourished AN. However, further mechanistic experiments will be needed to conclude on a causal role of the ghrelin/LEAP-2 balance in AN symptoms.

## STAR★Methods

### Key resources table

**Table:** 

REAGENT or RESOURCE	SOURCE	IDENTIFIER
**Chemicals, peptides, and recombinant proteins**		

p-hydroxymercuribenzoic-acid	Sigma-Aldrich	Cat#55540
EDTA Aprotinin Vacutainer tubes	Greiner Bio One SAS	Cat#454261
Microvette CB 300 K2 EDTA	Sarstedt, Nümbrecht	Cat#16.444.100

**Critical commercial assays**		

Acyl ghrelin enzyme immunoassay (human)	Bertin Bioreagents	Cat#A05106
Acyl ghrelin enzyme immunoassay (rat/mouse)	Bertin Bioreagents	Cat#A05317
Liver-Enzyme-Antimicrobial-Peptide-2 (human/mouse)	Phoenix pharmaceuticals	Cat#EK-075-40

**Experimental models: Organisms/strains**		

C57BL6/J female mice	Charles River	Not Applicable

**Other**		

ActiviWheel wheels and Software	Intellibio	Cat#A-1908-00150

### Resource availability

#### Lead contact

Further information and requests for resources and reagents should be directed to and will be fulfilled by the Lead Contact, Virginie Tolle (virginie.tolle@inserm.fr).

#### Materials availability

This study did not generate new unique reagents.

### Experimental model and study participant details

#### Animals

Seven weeks old female C57Bl6/J mice (Charles River Laboratories, L’Arbresle, France) were acclimatized for five days to the animal facility (Institute of Psychiatry and Neuroscience of Paris) upon their arrival. They were kept in a pathogen-free barrier facility on a 12h light-dark cycle (dark onset at 18:00) in an environment with controlled temperature (22°C-24°C) and stable humidity levels. Mice were fed on standard chow diet (3% fat, 16% protein, 60% carbohydrate, 4% fibers, 2.79 kcal/g; Safe A04) and had *ad libitum* access to purified water in their home cage. Body weight was assessed on weekdays during food restriction and refeeding. Food was delivered directly in the home-cage at 17:00 for all food restricted animals. All experiments were carried out according to the European Communities Council Directives (86/609/EEC) and approved by Regional Ethics Committee (CEEA.34) of Université Paris Cité. All experiments were lead on young female mice to be as close as possible to clinical situations.

#### Participants

##### Healthy controls

We recruited 5 young females aged 20 to 31 years old (24.6±1.60) who volunteered through local information procedures in student meetings and research institutes. Participants were healthy volunteers with no personal or family history of eating disorder and a body mass index between 18.5 and 25 kg/m^2^. Exclusion criteria were: a diagnosis of psychiatric disorder according to the DSM-5, debilitating medical condition, ongoing medication (except birth control pill), contraindication for fasting. The protocol was approved by a national Ethical Committee (A01636-49) and written informed consent was obtained for all participants.

##### Patients with anorexia nervosa (AN)

Thirty women aged from 19 to 53 years old (26.22±1.79), with DSM-5 criteria of AN, were included during their admission in a department specialized in eating disorders (CMME, GHU Paris Psychiatrie et Neurosciences). Twenty-nine patients went through the entire protocol so far, inclusion and follow-up are still ongoing. The protocol was approved by national Ethical Committee (CPP19.07.26.54412). Written informed consent was obtained for all participants on inclusion. All data were recorded anonymously.

### Method details

#### Preclinical model of food restriction in mice

##### Food restriction and progressive refeeding

To explore the effect of long-term food restriction and chronic running wheel activity, we used the Food Restriction and Wheel (FRW) protocol^25^ with small modifications. Mice were housed two per cage to limit external stress due to isolation and hypothermia and favor adequate food intake follow-up in each pair. During 5 days, animals were kept in pairs with free access to food to calculate *ad libitum* food intake of standard chow. *Ad libitum* food intake for each pair of animal was measured from daily weight of pellets in the feeder and used it as a baseline (100% *ad libitum* food intake) to calculate food restriction (FR). Animals were then randomized in four groups of eight mice according to their initial body weight (average body weight on arrival of 16.19 g ± 0.20 g). The groups “*ad libitum*” (AL) and “*ad libitum* and wheel” (ALW) were kept with free access to chow during the entire experiment. Mice of the groups “food restriction” (FR) and “food restriction and wheel” (FRW) were exposed to 30% food restriction for three days followed by a 50% food restriction for the next 12 days. After 15 days of FR, refeeding started on Day 15 with a progressive 10% increase of food intake every two days during 10 days (60% for 2 days, 70% for 2 days, 80% for 2 days, 90% for 2 days and then 100%) until Day 25. Food was delivered as pellets of standard chow directly in the home cage for both animals at the same time around 17:00. Cages of ALW and FRW group were equipped with free running wheels (diameter: 230mm; width: 50mm; 1 revolution = 0.72m) with automatic recording of their running wheel activity (ActiviWheel wheels and Software; Cat#A-1908-00150, Intellibio, Seichamps, France). The running wheel activity with wheel revolution and time spent running was recorded every 10 min. Running wheel activity was divided into nighttime activity (18:00 to 06:00) usual active phase for rodent, food anticipatory activity (13:30 to 17:00) described as an active phase in anticipation to food delivery in FR mice, and total activity on 24h. Data from running wheel activity were extracted with an Excel® macro to obtain cumulative activity data for these three time-periods and was used to validate the use of the running wheel by all animals from ALW and FRW groups. Cages of AL and FR groups were equipped with similar wheels locked with metal clamps in order to control for cage enrichment. Body weight was monitored every weekday during the protocol and blood glucose was measured three times per week to ensure tolerance to FR (FreeStyle Optium Neo, Abbott, The Netherlands).

##### Acute fasting and refeeding

Seven- weeks old female mice (n=7) went through a 48h fasting with complete withdrawal of standard chow at 10:00 on day 1. After 48h fasting (“fasted” group), a first blood sample was collected and refeeding started with *ad libitum* standard chow. A second blood sample was performed after 48h of refeeding (“Refed” group). Body weight and blood glucose were assessed at each time-point. Blood sample were performed between 10:00 and 12:00 (Figure S1).

#### Clinical study

##### Fasting and refeeding in healthy controls

Participants were asked to follow a 15-hours fast between 20:00 to 11:30, without ingestion of any food or liquids beside water (Figure S1). Participants arrived at the hospital at 11:00 and were measured and weighed in the fasted state. All participants received the same standard meal between 12:00 and 12:30, of around 600 kcal containing proteins, carbohydrates, fibers, and sugar, considered a standard caloric intake for a young woman during summer considering very high temperatures. A first blood sample was withdrawn in the fasted state before the meal and a second blood sample 90 min following the end of the meal.

##### Longitudinal study design in Anorexia Nervosa

This study constitutes the first analysis from an ongoing longitudinal study devoted to exploring the remission process in AN. All participants included had three visits i/ the first visit (V1), in an undernourished state, performed in the first week after admission of inpatients, ii/ the second visit (V2) took place after four months of intensive care and before hospital discharge when participants reach a target body mass index (BMI>19 kg/m^2^) therefore being considered as in a refed state, iii/ the third visit (V3) took place six months after discharge with an evaluation of the remission status (still present *versus* lost). The diagnosis of “stable” remission at V3 was proposed when patients had a stable BMI ≥ 18.5 kg/m^2^ six months after hospital discharge, remission being considered “unstable” otherwise. Each visit consisted of a clinical evaluation which included the assessment of weight, BMI, a blood sample for metabolic explorations, and a psychiatric evaluation with assessment of i/ AN subtype (Restrictive “AN-R”, or Binge Purge “BP”), ii/ eating disorder symptoms with Eating Disorder Inventory, EDI-2, iii/ physical activity with the Exercise Addiction Index (EAI).^38^

#### Blood sample collection, processing and storage

##### Preclinical studies

In order to measure acyl ghrelin (AG) and LEAP-2 concentrations at various nutritional status in mice, we performed blood collection during *i)* long-term food restriction (Days 13-14) and following progressive refeeding (Days 25-26, *i.e.* on the day of sacrifice) and *ii)* during 48h fasting and following 48h refeeding. The first blood sample was collected from the caudal vein on capillary tubes coated with EDTA (Microvette CB 300 K2 EDTA, Cat#16.444.100, Sarstedt, Nümbrecht, Germany) after a small scalpel incision on the tip of the tail in awake animals. Blood was then immediately treated with p-hydroxy-mercuribenzoic-acid (PHMB 0.4 mM final concentration) (Cat#16.444.100, Sigman-Aldrich, Saint-Quentin-Fallavier, France), a serine-protease inhibitor. Animals were then kept two days without blood sampling or manipulation to allow recovery. The second blood sample was collected at the end of the refeeding protocol (Day 25-26, *i.e.* on the day of sacrifice for the progressive refeeding or after 48h refeeding), through a cardiac puncture performed with an EDTA-coated 1-mL syringe (at a final concentration of EDTA 1 mg/ml in blood) on deeply anesthetized animals with a mix of ketamine and xylazin. Blood was then transferred in an Eppendorf tube and supplemented with PHMB (0.4 mM final concentration). Samples were kept on ice at 4°C and centrifuged at 4°C (1000xg for 10 min) in order to collect plasma. Two plasma samples were prepared, one as a standard aliquot and one immediately acidified aliquot with HCl (0.1N final concentration) to preserve ghrelin stability. Plasma samples were then frozen on dry ice and stored at -80°C until assays. All blood samples were collected between 14:00 and 17:00, therefore before food delivery.

##### Clinical studies

Blood was collected at each visit after an overnight fast on Vacutainer tubes treated with EDTA and Aprotinin (Cat#454261, Greiner Bio One SAS, Courtaboeuf, France). After collection, blood was kept at 4°C before centrifugation within 2h (1000xg for 10 min at 4°C). Plasma was aliquoted and one aliquot was immediately acidified with HCl (final concentration of 0.1N). Samples were stored at -80°C at Centre de Ressources Biologiques (CRB) of GHU Paris Psychiatrie et Neurosciences and assayed within 6 months.

#### ELISA immunoassays

Plasma concentration of acyl ghrelin (AG) was evaluated with specific enzyme-immunoassay kits for mice and humans (Cat#A05317 for mice and Cat#A05106 for human, Bertin Bioreagents, Montigny le Bretonneux, France). All used samples came from acidified aliquot as acidification is known to preserve ghrelin stability. External quality control of the same mice and human plasma was respectively used in all assays to ensure inter-assay stability. Intra- and inter- assay coefficients of variation were respectively of 6.1% and 5.7% in mice, and 9 and 16% in humans. Plasma concentrations of LEAP-2 were measured with enzyme-immunoassay kit (Cat#EK-075-40, Phoenix Pharmaceuticals, Burlingame, USA). The commercial kit used here recognizes both mouse and human LEAP-2, i.e. LEAP-2 (38-77) (Human) / LEAP-2 (37-76) (Mouse) (100% cross-reactivity). External quality control of the same mice and human plasma was respectively used in all assays to control inter-assay variation. Intra- and inter-assay coefficient of variation were respectively <10% and <15%.

Concentrations were given in pmol/L and the AG/LEAP-2 molar ratio was calculated using molar concentrations of AG and LEAP-2.

### Quantification and statistical analysis

Data are expressed as mean ± SEM except if otherwise specified. Paired t-test and repeated-measures ANOVA were used in preclinical and clinical studies after assessing data normality using Shapiro-Wilk or d’Agostino-Pearson tests. Log-transformation were performed on data to ensure normality of the results when necessary. Tukey’s post-hoc comparison were made when ANOVA was considered significant (p<0.05). We provide all the details about ANOVA results in Table S1 to facilitate reading. Non-parametric tests were used when appropriate. Correlations were calculated with linear regression. Multivariate adjustment to control for confounding variables was not applicable in this sample of limited size. We controlled for confounding factors between stable and unstable remission groups, comparing clinical data known to be associated with clinical outcome using a linear regression. Statistical tests used are indicated in figures legends. P value under 0.05 were considered significant. Statistical analyses and graphs were made with GraphPad Prism 9.0 (Abacus Concept, Berkeley, CA, USA).

### Additional resources

This work is part of a clinical trial registered as #NCT04560517 (URL: https://Clinicaltrials.gov/study/NCT04560517).

## Data Availability

• This study does not report original codes. • All data reported in this paper will be shared by the lead contact upon request. • Any additional information required to reanalyze the data reported in this paper is available from the lead contact upon request.
