# Supplementary material for: The role of dysregulated ghrelin/LEAP-2 balance in anorexia nervosa
Source: iScience. 2023 Sep 22;26(11):107996. doi: 10.1016/j.isci.2023.107996 (PMC10587521; doi:10.1016/j.isci.2023.107996)
Supplement: Document S1. Figures S1 and Tables S1–S3 [file mmc1.pdf]

iScience, Volume *26*

Supplemental information

The role of dysregulated ghrelin/LEAP-2 balance in anorexia nervosa

Chloé Tezenas du Montcel, Philibert Duriez, Jingxian Cao, Nicolas Lebrun, Nicolas Ramoz, Odile Viltart, Philip Gorwood, and Virginie Tolle

SUPPLEMENTAL INFORMATION

Figure	ANOVA	Mice per group	Factors	$F_{(DFn,DFd)}$	p-value
**Body Weight (g)**	Three-way	AL/ALW n=8	Time ****	$F_{(16,448)}$= 70,930	p<0,001
Figure 1A		FR/FRW n=8	FR ****	$F_{(1,28)}$= 73,060	p<0,001
			Running	$F_{(1,28)}$=0,580	p=0,452
			Time x FR ****	$F_{(16,448)}$=79,200	p<0,001
			Time x Running **	$F_{(16,448)}$=2,539	P<0,001
			FR x Running	$F_{(1,28)}$=2,059	p=0,162
			Time x FR x Running	$F_{(16,448)}$= 1,649	p=0,053
**AG in food restriction (pmol/L)**	Two-way	AL/ALW n=8	Running	$F_{(1,28)}$=0,386	p=0,539
Figure 1B		FR/FRW n=8	FR ****	$F_{(1,28)}$=40,630	p<0,001
			FR x Running	$F_{(1,28)}$=0,009	p=0,755
**LEAP-2 in food restriction (pmol/L)**	Two-way	AL/ALW n=8	Running	$F_{(1,28)}$=0,025	p=0,874
Figure 1C		FR/FRW n=8	FR ****	$F_{(1,28)}$=11,020	p=0,002
			FR x Running	$F_{(1,28)}$=0,011	p=0,917
**AG/LEAP-2 molar ratio in food restriction**	Two-way	AL/ALW n=8	Running	$F_{(1,28)}$=0,559	p=0,461
Figure 1D		FR/FRW n=8	FR ****	$F_{(1,28)}$=30,490	p<0,001
			FR x Running	$F_{(1,28)}$=0,651	p=0,426
**AG in refed mice (pmol/L)**	RM	FR n=8	Refeeding ****	$F_{(1,13)}$=26,370	p<0,001
Figure 1E		FRW n=7	Running	$F_{(1,13)}$=1,310	p=0,272
			Refeeding x Running	$F_{(1,13)}$=0,007	p=0,936
**LEAP-2 in refed mice (pmol/L)**	RM	FR n=8	Refeeding ****	$F_{(1,14)}$=17,630	p<0,001
Figure 1F		FRW n=8	Running	$F_{(1,14)}$=0,377	p=0,055
			Refeeding x Running	$F_{(1,14)}$=1,458	p=0,247
**AG/LEAP-2 molar ratio in refed mice**	RM	FR n=8	Refeeding ****	$F_{(1,13)}$=20,530	p<0,001
Figure 1G		FRW n=8	Running	$F_{(1,13)}$=0,193	p=0,668
			Refeeding x Running	$F_{(1,13)}$=1,041	p=0,326

Table S1. ANOVA table of preclinical data, related to Figure 1. Data represent mean±SEM and were analyzed using three-way ANOVA (A), two-way ANOVA (B-D) or RM ANOVA (E-G).

*** $p<0.001$, **** $p<0.0001$. AG: Acyl-ghrelin. LEAP-2: Liver-expressing Antimicrobial Peptide-2.

	V1 (n=30)	V2 (n=30)	Paired Student t-test
**Age (years)**	27,00±1,68	-	
**Duration of illness (months)**	92,27±17,70	-	
**Days before inclusion**	4,43±0,44	-	
**Subtype**			
AN-R	77,0% (n=23)	-	
AN-BP	23,3% (n=7)	-	
**BMI (kg/m2) ******	14,22±0,24	20,11±0,09	t=26,6 df=29 p<0,0001
**EDI-2 ******	90,64±9,00	62,94±7,73	t=5,21 df=29 p<0,0001
**EAI ****	25,61±2,36	21,51±2,04	t=2,90 df=29 p<0,01
**Weekly Leisure Activity (hour) ***	44,94±5,99	30,76±3,19	t=2,56 df=29 p=0,01
**AG (pmol/L) ******	36,22±5,36	20,79±3,88	t=5,47 df=29 p<0,0001
**LEAP-2 (pmol/L) ***	3830±390,60	3095±303,60	t=2,06 df=29 p<0,05
**AG/LEAP-2 molar ratio****	0,01±0,0001	0,007±0,0009	t=3,07 df=29 p<0,01

Table S2. Epidemiological, clinical, and biological data of 30 patients in the longitudinal follow-up study, related to Figure 3. Data represent mean±SEM and were analyzed using paired t-test: *p<0.05, **p<0.01, ****p<0.0001. AN-R: Anorexia Nervosa Restrictive subtype, AN-BP: Anorexia Nervosa, Binge Purge subtype. BMI: Body Mass Index. EDI-2: Eating Disorder Inventory-2, EAI: Exercise Addiction Index. AG: Acyl-ghrelin, LEAP-2: Liver Expressing Antimicrobial Peptide-2.

	Stable remission	Unstable Remission	Student or Mann-Whitney test
**N**	12	17	
**Age (years)**	25.58±1.63	28,00±2,76	p=0,879
**Duration of illness (months)**	96,67±27,62	94,35±24,83	p=0,939
**BMI (kg/m2)** V1	14,66±0,35	13.9±0.33	p=0,140
V2*	20,25±0,09	20.01±0.15	p=0,043
V3****	20.07±0.44	16.58±0.35	p<0,001
**Weight gain (% initial body weight)**	39.23±4.21	45.08±2.51	p=0,27
**EDI-2** V1	111.1±15.25	81.1±12.13	p=0,136
V2	77.67±14.69	57.00±10.23	p=0,234
V3	80.33±15.97	38.60±15.63	p=0,053
**EAI** V1	30.83±4.88	21.06±2.58	p=0,085
V2	23.88±4.78	19.93±2.07	p=0,70
V3	22.83±3.00	13.55±3.01	p=0,061
**AG (pmol/L)** V1	31.99±7.88	39.96±7.71	p=0,444
V2	19.05±6.47	22.09±5.17	p=0,491
V1 versus V2 in stable **			p=0,009
V1 versus V2 in unstable ***			p<0,001
**LEAP-2 (pmol/L)** V1	3469±672.3	4202±490.1	p=0,131
V2	3291±506.1	3021±405.1	p=0,678
V1 versus V2 in stable			p=0,640
V1 versus V2 in unstable *			p=0,044
**AG/LEAP-2 molar ratio** V1	0.011±0.003	0.011±0.002	p=0,913
V2	0.006±0.001	0.008±0.001	p=0,394
V1 versus V2 in stable *			p=0,043
V1 versus V2 in unstable			p=0,078

Table S3. Epidemiological, clinical and biological data on patients evaluated 6 months after discharge, related to Figure 4. Patients were characterized with either stable remission

(n=12) if BMI≥18,5 or unstable remission (n=17) if BMI<18,5. Data represent mean±SEM and were analyzed using Student t-test or non-parametric Mann Whitney test depending on data distribution. *p<0.05, **p<0.01, *** p<0.001, **** p<0,0001. BMI: Body mass Index (kg/m^{2}), AG: Acyl-ghrelin. LEAP-2: Liver-expressing Antimicrobial Peptide-2. V1: undernourished state at admission; V2: refed state at discharge.

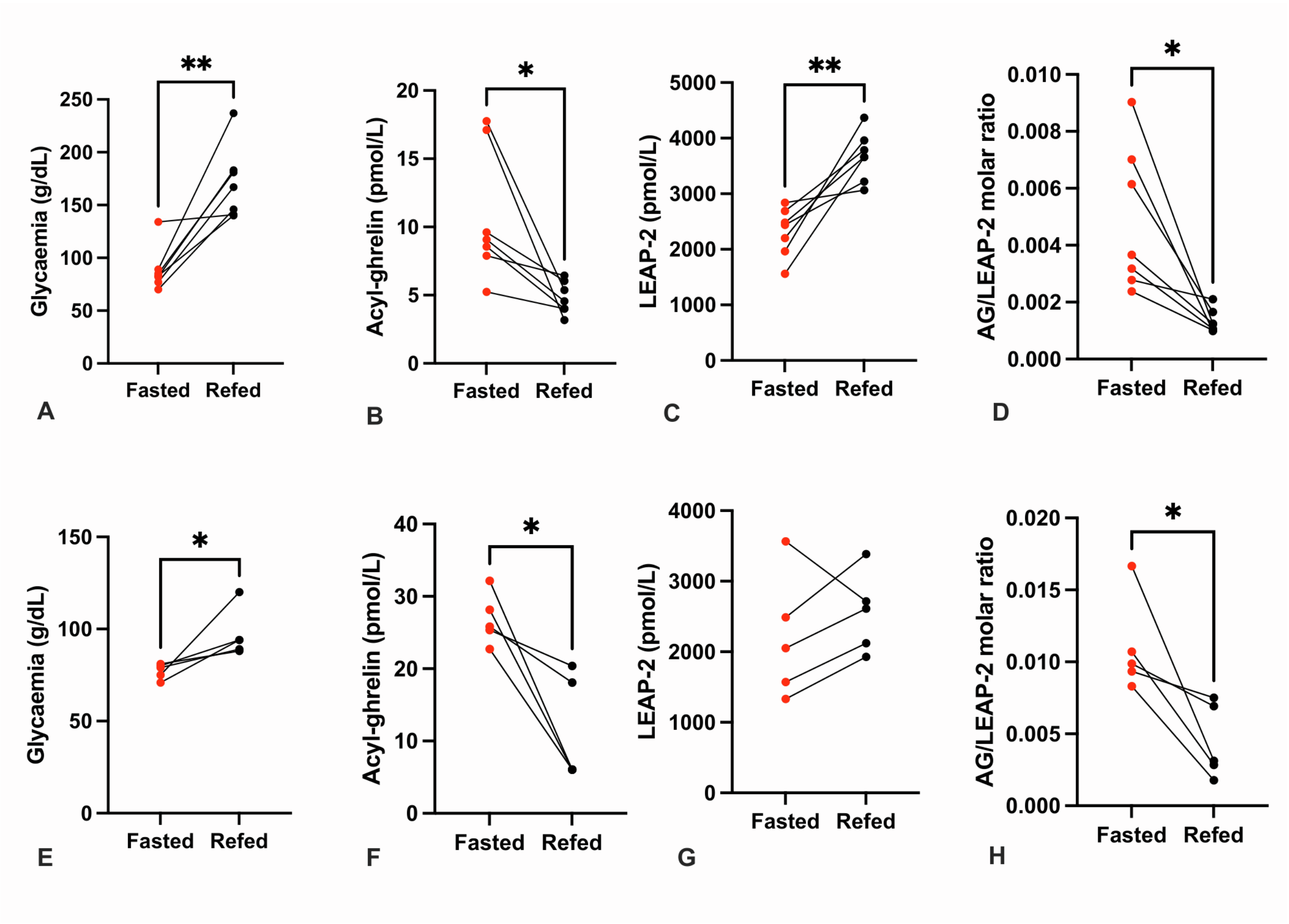

Figure S1. Longitudinal changes in plasma AG and LEAP-2 levels during acute fasting and refeeding in eight-week-old female mice (A-D) and in healthy women (E-H), related to the STAR Methods. Acute 48h fasting in female significantly decreased blood glucose levels (A) that was restored after 48h *ad libitum* refeeding. Plasma levels of AG were significantly decreased (B) but LEAP-2 levels (C) were increased with refeeding. AG/LEAP-2 molar ratio (D) was significantly higher in fasted compared to refed animals. Blood glucose and plasma AG and LEAP-2 levels were assayed in five healthy women after a 15-hours fast and 2 hours after a standard meal. Plasma levels of glucose (E) increased after a meal and AG (F) decreased. LEAP-2 levels increased after the meal in four participants out of five. AG/LEAP-2 molar ratio (H) were significantly higher in fasted compared to post-prandial state in humans. Data are presented as individual values. AG: Acyl-ghrelin. LEAP-2: Liver-expressing Antimicrobial Peptide-2. Statistical analysis: Paired t-test. *p<0.05, ** p<0.01.
